# Dynamic changes of the extracellular matrix after acute tako-tsubo cardiomyopathy

**DOI:** 10.1186/1532-429X-17-S1-P259

**Published:** 2015-02-03

**Authors:** Trevor Ahearn, Baljit Jagpal, Donnie Cameron, Bernice K Ng, Caroline Scally, David M Higgins, John Horowitz, Michael P Frenneaux, Dana K Dawson

**Affiliations:** Medical Physics, NHS Grampian, Aberdeen, UK; ABIC, Aberdeen University, Aberdeen, UK; Cardiology, Aberdeen University, Aberdeen, UK; Philips Healthcare, Guildford, UK; Cardiology, Queen Elizabeth Hospital, Adelaide, SA Australia

## Background

We have recently demonstrated that cardiac energetic impairment and global myocardial edema persists for at least 4 months after an acute episode of Tako-tsubo cardiomyopathy (TTC). The aim of the current study was to evaluate the regional edema acutely and the status of the extracellular matrix at follow up

## Methods

Eleven patients (10F, mean age 56±16yrs) with a clear diagnosis of ST-elevation TTC and emotional trigger were prospectively enrolled and underwent cardiac magnetic resonance acutely (day 0-3) and after 4 months on a Philips 3T Achieva scanner. Native 3-3-5 (MOLLI) T1 mapping was applied acutely, and both native and post-contrast T1 mapping were performed at 4 months follow-up. Eleven healthy controls underwent only native T1 mapping. T1 maps were: generated using in-house software - written in IDL (Exelis. Boulder CO, USA); quality controlled with chi-square maps; and imported into Segment (Medviso, Lund University, Sweden), where T1 values were generated for 16 segments. Extracellular volumes (ECV) were calculated for the follow-up scan using:

ECV=(1-hermatocrit)(ΔR_1myocardium_/ ΔR_1blood_)

Segments were grouped according to their wall motion (WM) on the acute scan (normal/abnormal).

## Results

From the acute to the follow-up scan, the LVEF improved from 54±12% to 66±6%, whereas LV mass index decreased from 77±15 g/m^2^ to 68±14 g/m^2^, both p<0.05.

*At the acute scan*, native T1 of abnormal WM segments was significantly longer compared with T1 from normal WM segments (1270±95 vs 1225±43 ms, p<0.05) and both were significantly increased compared to healthy controls (1188±16, p<0.05).

*At the follow-up scan*, ECV was increased to a similar extent both in segments that were dysfunctional and those that were normally contracting in the acute phase (33% and 34% respectively, p=0.05).

## Conclusions

We demonstrate oedema in both normal and abnormally contracting segments in patients with acute TTC and a similar degree of extracellular expansion at follow-up.

## Funding

Tenovus Scotland. Grant number G13/10.

